# SARS-CoV-2 spike protein S1 activates Cx43 hemichannels and disturbs intracellular Ca^2+^ dynamics

**DOI:** 10.1186/s40659-023-00468-9

**Published:** 2023-10-25

**Authors:** Juan Prieto-Villalobos, Claudia M. Lucero, Maximiliano Rovegno, Gonzalo I. Gómez, Mauricio A. Retamal, Juan A. Orellana

**Affiliations:** 1 Departamento de Neurología, Escuela de Medicina and Centro Interdisciplinario de Neurociencias, Facultad de Medicina, Pontificia Universidad Católica de Chile, Marcoleta 391, Santiago, Chile; 2 Institute of Biomedical Sciences, Faculty of Health Sciences, Universidad Autónoma de Chile, Santiago, Chile; 3 Departamento de Medicina Intensiva, Facultad de Medicina, Pontificia Universidad Católica de Chile, Santiago, Chile; 4 Programa de Comunicación Celular en Cancer, Facultad de Medicina Clínica Alemana, Universidad del Desarrollo, Santiago, Chile

**Keywords:** Hemichannels, COVID-19, Connexin 43, SARS-CoV-2, ACE2, ATP and spike S1

## Abstract

**Background:**

Severe acute respiratory syndrome coronavirus 2 (SARS-CoV-2) causes the ongoing coronavirus disease 2019 (COVID-19). An aspect of high uncertainty is whether the SARS-CoV-2 per se or the systemic inflammation induced by viral infection directly affects cellular function and survival in different tissues. It has been postulated that tissue dysfunction and damage observed in COVID-19 patients may rely on the direct effects of SARS-CoV-2 viral proteins. Previous evidence indicates that the human immunodeficiency virus and its envelope protein gp120 increase the activity of connexin 43 (Cx43) hemichannels with negative repercussions for cellular function and survival. Here, we evaluated whether the spike protein S1 of SARS-CoV-2 could impact the activity of Cx43 hemichannels.

**Results:**

We found that spike S1 time and dose-dependently increased the activity of Cx43 hemichannels in HeLa-Cx43 cells, as measured by dye uptake experiments. These responses were potentiated when the angiotensin-converting enzyme 2 (ACE2) was expressed in HeLa-Cx43 cells. Patch clamp experiments revealed that spike S1 increased unitary current events with conductances compatible with Cx43 hemichannels. In addition, Cx43 hemichannel opening evoked by spike S1 triggered the release of ATP and increased the [Ca^2+^]_i_ dynamics elicited by ATP.

**Conclusions:**

We hypothesize that Cx43 hemichannels could represent potential pharmacological targets for developing therapies to counteract SARS-CoV-2 infection and their long-term consequences.

**Supplementary Information:**

The online version contains supplementary material available at 10.1186/s40659-023-00468-9.

## Background

Severe acute respiratory syndrome coronavirus 2 (SARS-CoV-2) causes the ongoing coronavirus disease 2019 (COVID-19). Since its first detection in late 2019 (Wuhan, China), SARS-CoV-2 has persistently and seriously challenged global human health and economy [1]. The spread of SARS-CoV-2 is usually through the droplet spray produced by coughing, sneezing, and talking. Most people with COVID-19 are either asymptomatic or experience mild symptoms, including anosmia (loss of smell), fever, fatigue, headache, cough, muscle aches, and loss of appetite [2]. Nevertheless, in specific susceptible populations, SARS-CoV-2 infection can develop into a severe type of pneumonia which, if not adequately treated, could lead to acute respiratory distress syndrome, a life-threatening form of respiratory failure [3]. Predictive models estimate that SARS-CoV-2 infection has been implicated in 18.2 million deaths worldwide [4].

Although most individuals fully recover from COVID-19, some people experience the persistence of symptoms beyond three months of SARS-CoV-2 infection, lasting at least two months [5]. This condition has been referred to as “long-COVID” or “post-acute COVID syndrome” and commonly includes fatigue, breathlessness, post-exertional malaise, “brain fog”, headaches, nausea, vomiting, anxiety, depression, skin rash, joint pain, and palpitations [6, 7]. Long-COVID does not rely on persistent infection and develops regardless of the severity of the initial symptoms [8]. It is not yet known how persistent symptoms of long-COVID will last, but recent studies suggest that it may depend on antigen persistence and sustained specific immune responses to SARS-CoV-2 [9]. This background positions long-COVID as the “next health disaster” worldwide [10].

An aspect of high uncertainty is whether the SARS-CoV-2 *per se* or the systemic inflammation induced by viral infection directly affects cellular function and survival in different tissues [11]. In this context, it has been postulated that tissue dysfunction and damage observed in COVID-19 patients may rely on the direct effects of SARS-CoV-2 viral proteins [12–15]. Structurally, SARS-CoV-2 is a linear, positive-sense, single-stranded RNA-enveloped virus formed by four proteins: the spike, envelope, membrane, and nucleocapsid proteins [16]. The spike protein allows the virus to infect mammalian host cells by engaging the angiotensin-converting enzyme 2 (ACE2) receptor. Two monomeric subunits, S1 and S2, comprise the spike protein. The S1 subunit contains the binding domain that attaches to the ACE2 receptor. In contrast, the S2 subunit mediates the fusion of viral and cell membranes via its cleavage by diverse host proteases, including transmembrane serine protease 2 and cathepsins [17]. The latter eventually could produce the detachment of the S1 subunit (or spike protein S1 [spike S1]) and its release into the interstitium and blood plasma, from where it can reach different tissues, including the nervous system [12, 13, 18]. Serum levels of spike S1 correlate with the severity of COVID-19 disease [19] and are higher in individuals with ongoing long-COVID [20]. The spike S1 causes endothelial dysfunction [21, 22] and activates the complement system leading to platelet aggregation [23]. This viral protein disrupts the function of human cardiac pericytes [24] and the blood-brain barrier [25], whereas it also induces cognitive deficit and anxiety-like behaviors *in vivo* [14]. With this in mind, it has been proposed that acute and long-lasting symptoms of COVID-19 patients may originate from the direct action of spike S1 [12]. However, the mechanisms by which this viral protein could disturb cellular function remain to be fully elucidated.

Multiple studies have demonstrated that dysregulation of connexin hemichannel signaling contributes to cellular dysfunction with potentially detrimental consequences for tissue and organ homeostasis [26–31]. Connexin hemichannels belong to a family of large-pore channels formed by the transmembrane proteins called connexins [32]. Six monomers of these proteins oligomerize around a central pore to form a connexon or connexin hemichannel, which allows the ionic and molecular exchange between the cytoplasm and the extracellular space [33]. Within this family of channels, those formed by the connexin 43 (Cx43) are the most ubiquitous and play essential roles in the autocrine/paracrine signaling in various organs and systems [34–37]. Indeed, the diffusion of paracrine biomolecules and ions through Cx43 hemichannels, including Ca^2+^, ATP, PGE_2_, glucose and glutamate, contributes to the coordination and synchronization of Ca^2+^ wave propagation [38, 39], cell migration and proliferation [40, 41], cardiac excitability [42], synaptic transmission [43, 44] and memory consolidation [45–47]. Nevertheless, under pathological conditions, the persistent and exacerbated opening of connexin hemichannels causes the release of potentially harmful molecules (e.g., ATP, glutamate), cytosolic Ca^2+^ ([Ca^2+^]_i_) overload and/or loss of ionic and osmotic balance [27, 30, 48].

Previous research has found a link between the activation of connexin hemichannels and virus infection. For instance, the human immunodeficiency virus (HIV) and its envelope protein (gp120) increase the activity of Cx43 hemichannels with negative repercussions for cellular function and survival [49, 50]. However, it is still unknown whether SARS-CoV-2 or its viral proteins, including spike S1, could impact the function of Cx43 hemichannels. Here, we demonstrate that spike S1 augments the activity of Cx43 hemichannels, this response being potentiated by ACE2 receptors. Moreover, this viral protein distinctively modulated ATP-mediated [Ca^2+^]_i_ dynamics depending on the presence of Cx43 and/or ACE2 receptors.

## Results

### Spike S1 increases the activity of Cx43 hemichannels

**Fig. 1:**
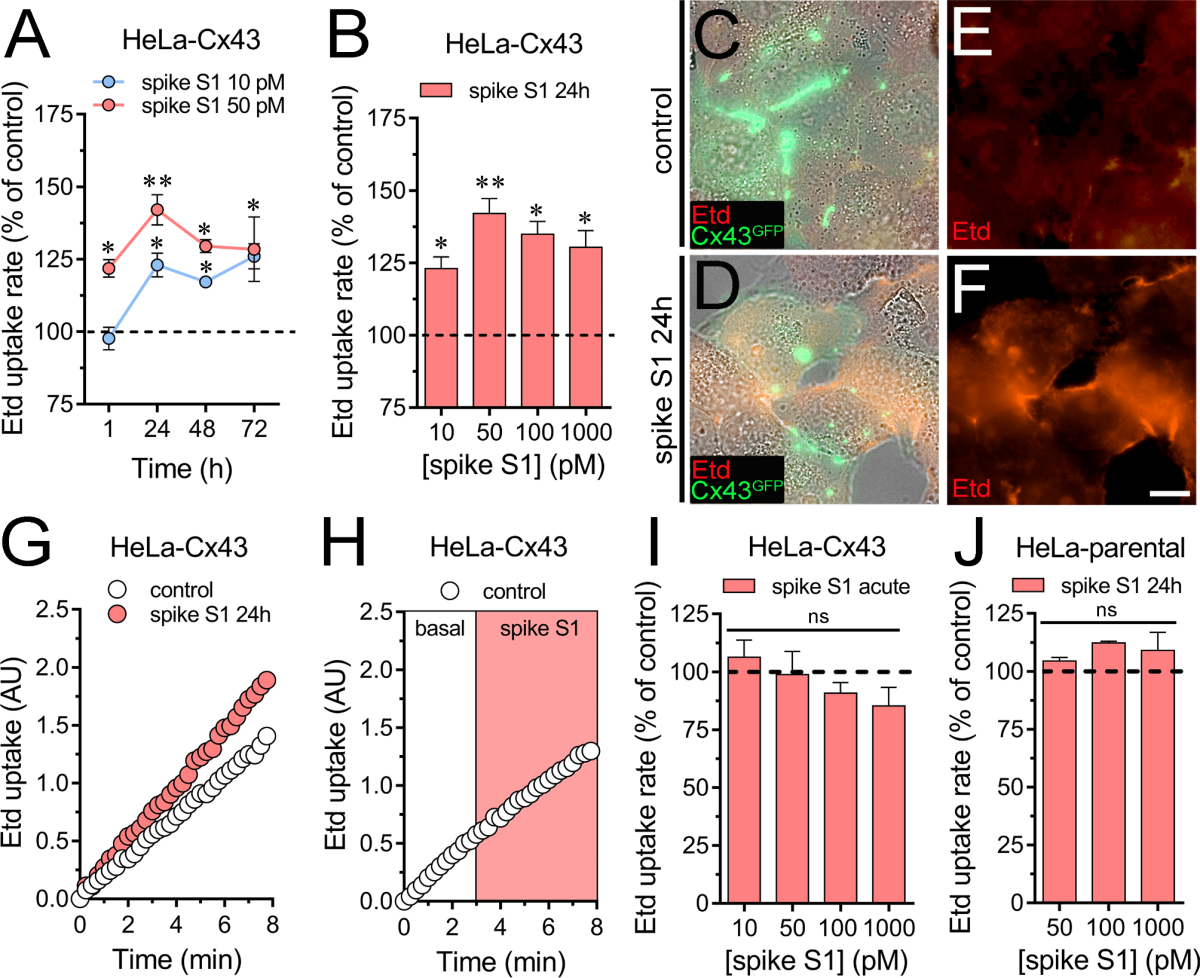
Spike S1 augments the activity of Cx43 hemichannels. (**A**) Averaged Etd uptake rate normalized with the control condition (dashed line) by HeLa-Cx43^GFP^ cells treated for several periods with 10 pM (blue circles) or 50 pM (red circles) spike S1. *p < 0.05, **p < 0.01; effect of spike S1 compared to control (two-way ANOVA followed by Tukey’s post-hoc test). (**B**) Averaged Etd uptake rate normalized with the control condition (dashed line) by HeLa-Cx43^GFP^ cells treated for 24 h with different concentrations of spike S1. *p < 0.05, **p < 0.01; effect of spike S1 compared to control (one-way ANOVA followed by Tukey’s post-hoc test). (**C-D**) Representative images depicting the phase view merged with Cx43^GFP^ (green) and Etd (red, 5 µM and 10 min of exposure) labeling by HeLa-Cx43^GFP^ cells under control conditions (**C**) or treated with 50 pM spike S1 for 24 h (**D**). (**E-F**) Etd staining by HeLa-Cx43^GFP^ cells showed in C and D. (**G**) Time-lapse measurements of Etd uptake by HeLa-Cx43^GFP^ cells under control conditions (white circles) or treated with 50 pM spike S1 for 24 h (red circles). (**H**) Time-lapse measurements of Etd uptake by HeLa-Cx43^GFP^ cells under control conditions acutely stimulated with 50 pM spike S1. Etd fluorescence was recorded in basal conditions for 3 min (white outline) and then an acute stimulation was performed during the following 5 min (red outline). (**I**) Averaged Etd uptake rate normalized with the control condition (dashed line) by HeLa-Cx43^GFP^ cells acutely stimulated with different concentrations of spike S1. (**J**) Averaged Etd uptake rate normalized with the control condition (dashed line) by HeLa-parental cells treated for 24 h with different concentrations of spike S1. Data were obtained from at least three independent experiments with three or more repeats each (≥ 20 cells analyzed for each repeat). Calibration bar = 12 μm

Previous studies have demonstrated that diverse pathogens augment the function of Cx43 hemichannels, including the HIV virion [49], the envelope HIV protein gp120 [50], lipopolysaccharide [51–53] and peptidoglycan [54]. With this in mind, we examined whether spike S1 could affect the function of Cx43 hemichannels expressed in HeLa cells. These cells transfected with Cx43 alone or tagged with fluorescent proteins express hemichannels at the cell surface, through which they take up and release small molecules, including dyes commonly used to assay hemichannel activity [55–57]. Thus, the functional state of Cx43 hemichannels was studied by recording the uptake of ethidium (Etd) in HeLa cells expressing Cx43 tagged with GFP (HeLa-Cx43^GFP^). Etd is a probe that crosses the plasma cell membrane by diffusing through large-pore channels such as hemichannels [58]. Once in the intracellular space, it binds to nucleic acids, emitting fluorescence equivalent to the function or activity of hemichannels. We found that spike S1 elicited a significant time-dependent increase in Etd uptake that peaked following 24 h of treatment in HeLa-Cx43^GFP^ cells (Fig. 1A-G). Moreover, this response was concentration-dependent, reaching the highest value with 50 pM spike S1 that gradually declined over greater concentrations (Fig. 1B). In contrast, acute incubation with spike S1 failed to elevate Etd uptake in HeLa-Cx43^GFP^ cells (Fig. 1H and **I**). On the other hand, the stimulus with spike S1 (50-1000 pM) for 24 h did not alter the Etd uptake in HeLa-parental cells, revealing that spike S1 increases specifically the activity of Cx43 hemichannels in HeLa cells (Fig. 1J).

### ACE2 potentiates spike S1-induced activity of Cx43 hemichannels

**Fig. 2:**
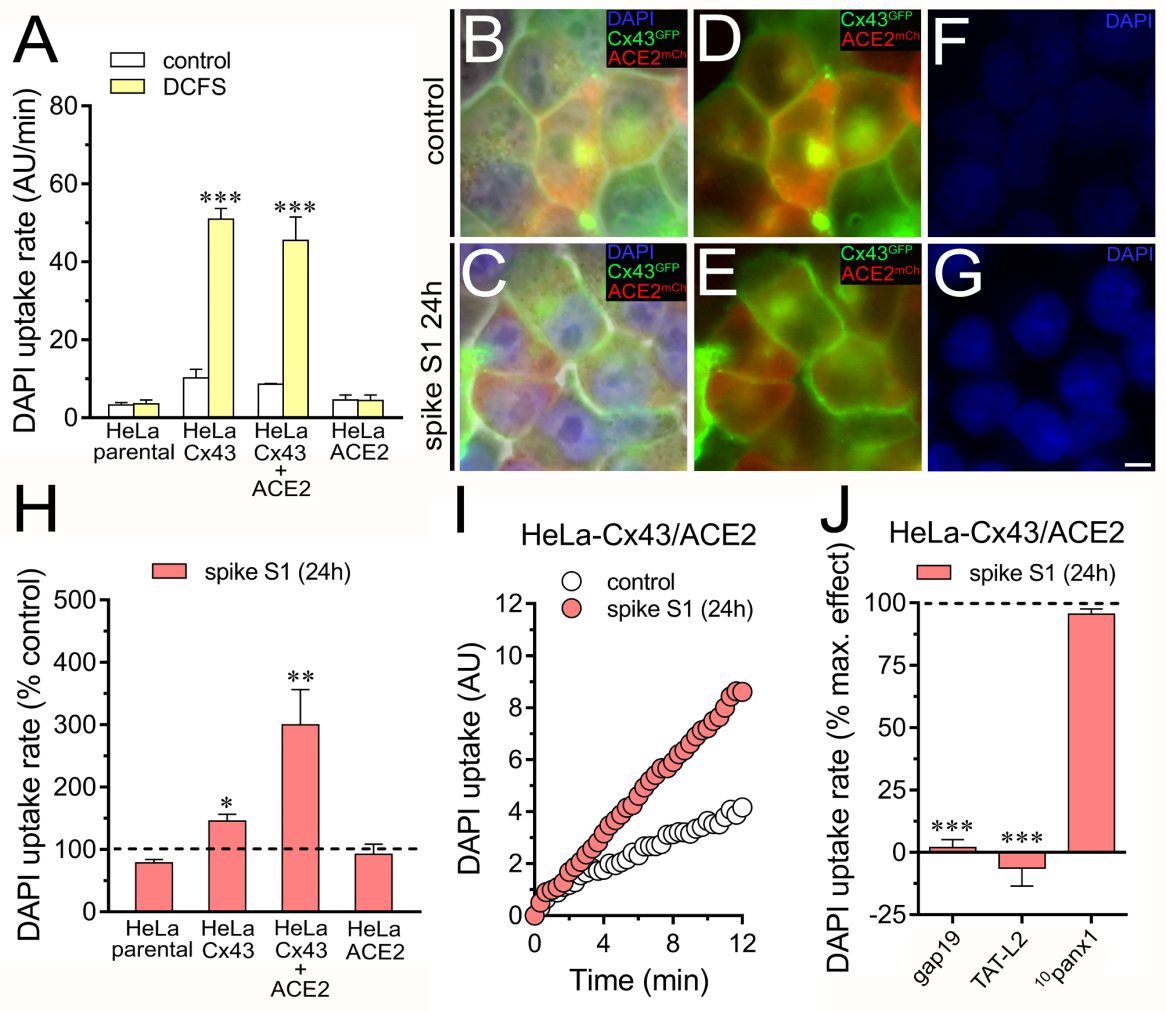
The activation of Cx43 hemichannels induced by spike S1 is potentiated by ACE2. (**A**) Averaged DAPI uptake rate by HeLa-parental, HeLa-Cx43^GFP^, HeLa-Cx43^GFP^/ACE2^mCherry^ or HeLa-ACE2^mCherry^ cells bathed with normal saline (control, white bars) or a divalent cation-free solution (DCFS, yellow bars). ***p < 0.001; effect of DCFS compared to control (two-way ANOVA followed by Tukey’s post-hoc test). (**B-C**) Representative images depicting the phase view merged with Cx43^GFP^ (green), ACE2^mCherry^ (red), and DAPI (blue, 10 µM and 10 min of exposure) labeling by HeLa-Cx43^GFP^/ACE2^mCherry^ cells under control conditions (**B**) or treated with 50 pM spike S1 for 24 h (**C**). (**D-E**) Cx43^GFP^ and ACE2^mCherry^ labeling by Cx43^GFP^/ACE2^mCherry^ cells showed in **B** and **C**. (**F-G**) DAPI staining by Cx43^GFP^/ACE2^mCherry^ cells shown in **B** and **C**. (**H**) Averaged DAPI uptake rate normalized with the control condition (dashed line) by HeLa-parental, HeLa-Cx43^GFP^, HeLa-Cx43^GFP^/ACE2^mCherry^ or HeLa-ACE2^mCherry^ cells treated with 50 pM spike S1 for 24 h. *p < 0.05, **p < 0.01; effect of spike S1 compared to control (one-way ANOVA followed by Tukey’s post-hoc test). (**I**) Time-lapse measurements of DAPI uptake by HeLa-Cx43^GFP^/ACE2^mCherry^ cells under control conditions (white circles) or treated with 50 pM spike S1 for 24 h (red circles). (**J**) Averaged DAPI uptake rate normalized to the maximum effect evoked by spike S1 (dashed line) by HeLa-Cx43^GFP^ cells acutely stimulated with different concentrations of spike S1. (**J**) Averaged DAPI uptake rate normalized with the control condition (dashed line) by HeLa-Cx43^GFP^/ACE2^mCherry^ cells treated with 50 pM spike S1 for 24 h plus gap19 (100 µM), TAT-L2 (200 µM) or ^10^panx1 (100 µM). ***p < 0.001; effect of spike S1 vs. blockers (one-way ANOVA followed by Tukey’s post-hoc test). Data were obtained from at least three independent experiments with three or more repeats each (≥ 20 cells analyzed for each repeat). Calibration bar = 8 μm

One widely accepted fact is that the spike S1 subunit possesses a receptor-binding domain that identifies ACE2 as its receptor [1, 59]. To examine whether ACE2 could contribute to the spike S1-induced activity of Cx43 hemichannels, we transfected HeLa cells, which lack endogenous ACE2 expression [1], with Cx43^GFP^ plus the human ACE2 tagged with mCherry (HeLa-Cx43^GFP^/ACE2^mCherry^). Because the mCherry tag of ACE2 impeded us from performing dye uptake experiments with Etd (due to similar emission wavelengths), we used DAPI, another well-known tracer employed for measuring Cx43 hemichannel activity [56, 60]. Stimulation with a divalent cation-free solution (DCFS) increases Cx43 hemichannel opening within seconds [57]; thereby, we used this condition to explore the status of DAPI uptake in Hela-Cx43^GFP^/ACE2^mCherry^ cells. Both Hela-Cx43^GFP^ and Hela-Cx43^GFP^/ACE2^mCherry^ cells showed a ~ 4.5-fold increase in DAPI uptake when stimulated with a DCFS (Fig. 2A). As expected, DCFS conditions did not augment DAPI uptake in Hela-parental or Hela-ACE2^mCherry^ cells, showing that DCFS-mediated membrane permeabilization to DAPI requires the presence of Cx43 (Fig. 2A). Similar to that observed with Etd, treatment with 50 pM spike S1 for 24 h resulted in a significant increase in DAPI uptake in both HeLa-Cx43^GFP^ and Hela-Cx43^GFP^/ACE2^mCherry^ cells (Fig. 2B-H). These responses were more prominent in Hela-Cx43^GFP^/ACE2^mCherry^ cells than HeLa-Cx43^GFP^ cells but not observed in Hela-parental or HeLa-ACE2 ^mCherry^ cells (Fig. 2H).

**Fig. 3:**
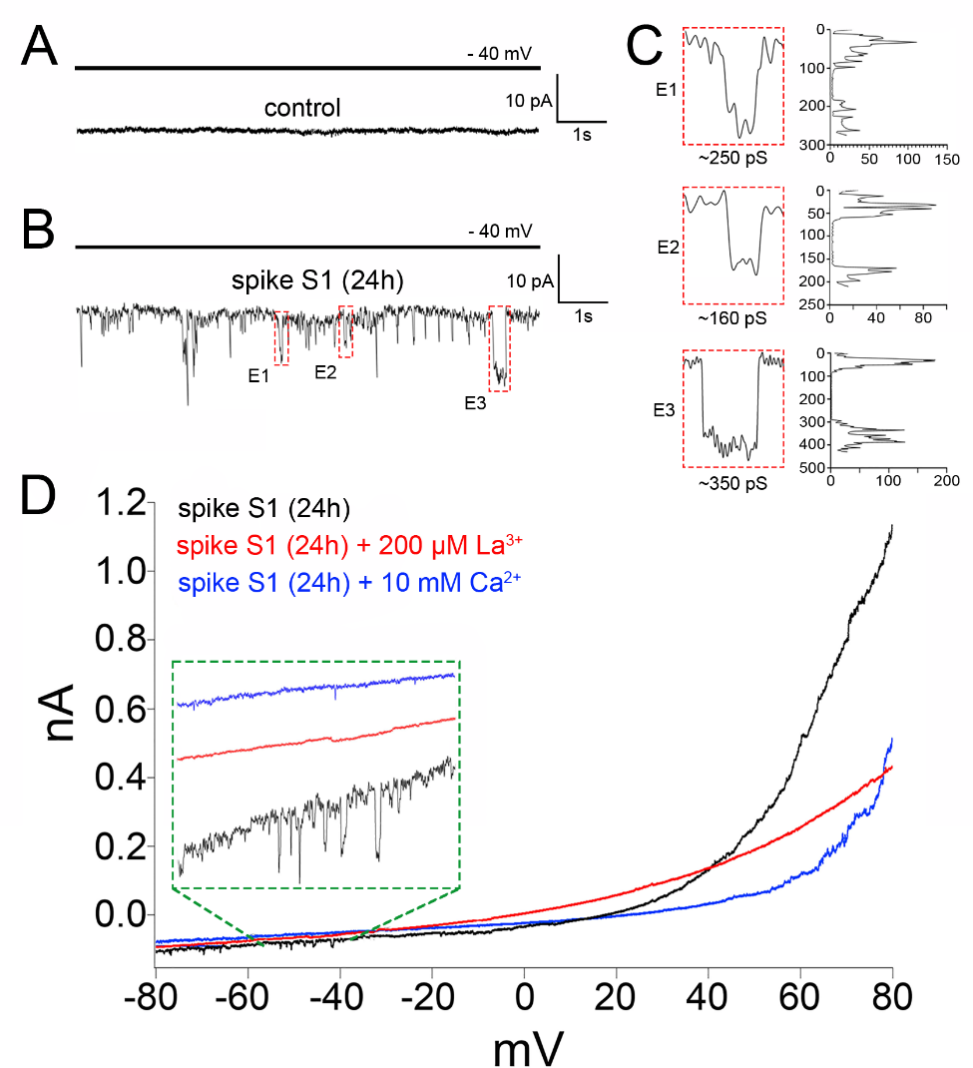
Spike S1 augments ionic currents associated to Cx43 hemichannels. (**A-B**) Whole-cell voltage-clamp recordings at -40 mV in HeLa-Cx43^GFP^/ACE2^mCherry^ cells under control conditions (**A**) or treated with 50 pM spike S1 for 24 h (**B**). (**C**) Expanded view of unitary current events depicted in the green squares (E1-E3) of the current trace in **B**. The frequency (counts) distribution of conductance values is also shown, corresponding to ∼250 pS, ∼160 pS and ∼350 pS. (**D**) Voltage ramps from − 80 to + 80 mV in HeLa-Cx43^GFP^/ACE2^mCherry^ cells treated with 50 pM spike S1 for 24 h alone (black line), in combination with 200 µM La^3+^ (red line) or plus 10 mM extracellular Ca^2+^ (blue line)

Next, we employed a pharmacological approach to support the involvement of Cx43 hemichannels in the spike S1-mediated DAPI uptake. Thus, gap19 (100 µM) or TAT-L2 (100 µM), two inhibitory mimetic peptides with sequences equivalent to intracellular L2 loop regions of Cx43 [61, 62]; completely suppressed the spike S1-induced DAPI uptake in Hela-Cx43^GFP^/ACE2^mCherry^ cells (Fig. 2J). DAPI is capable of penetrating the plasma membrane by passing through hemichannels formed by pannexin-1 (Panx1) [63], which are large-pore channels that share some of the characteristics and functions of connexin hemichannels [64]. Since Panx1 hemichannels were recently reported to be activated by the SARS-CoV-2 virus, we seek to rule out their participation in the uptake of DAPI induced by the spike S1 [65]. ^10^panx1 (100 µM), a blocking mimetic peptide with an amino acid sequence homologous to the first extracellular loop domain of Panx1 [66] failed to reduce the DAPI uptake evoked by spike S1 in Hela-Cx43^GFP^/ACE2^mCherry^ cells (Fig. 2J). To confirm the stimulatory effect of spike S1 on Cx43 hemichannel activity, we conducted whole-cell voltage-clamp experiments and recorded macroscopic membrane currents in Hela-Cx43^GFP^/ACE2^mCherry^ cells. Unitary current transitions were nearly undetectable at resting potentials in control Hela-Cx43^GFP^/ACE2^mCherry^ cells (Fig. 3A). However, the treatment with 50 pM spike S1 for 24 h induced several discrete unitary current events with conductances close to ~ 160 pS, ~ 250 pS or ~ 350 pS (Fig. 3B and **C**). These subconductances agree with those reported for GFP-Cx43 hemichannels expressed in C6 and HEK293 cells or reconstituted in planar lipid bilayers [67–69]. Consistent with this, the macroscopic currents triggered by spike S1 were significantly inhibited by the hemichannel blocker La^3+^ (200 µM) and did not occur when high extracellular Ca^2+^ was added to the bath solution (Fig. 3D). The latter is a well-known condition that reduces the open probability of Cx43 hemichannels [69]. Overall, these findings indicate that spike S1 boosts the activity of Cx43 hemichannels, this response being potentiated by ACE2.

### Spike S1 reduces gap junctional communication

**Fig. 4:**
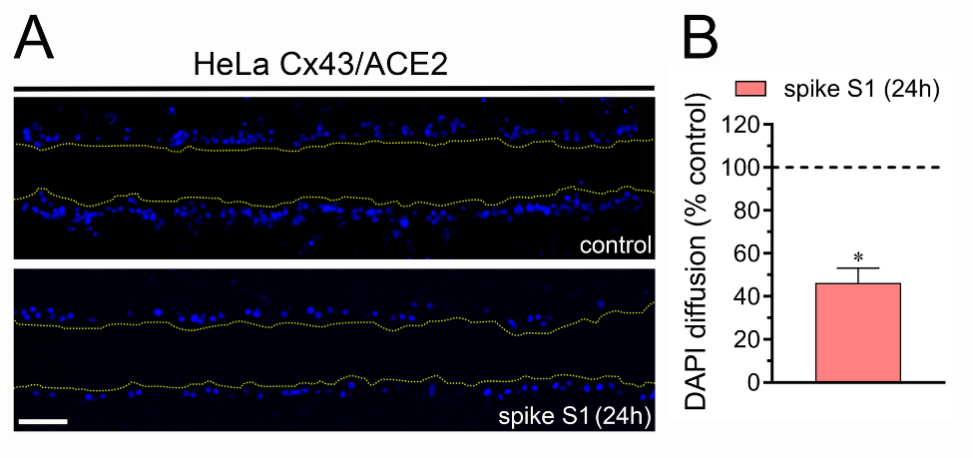
Spike S1 decreases gap junction channel activity. (A) Representative fluorescence micrographs of SL/DT with DAPI by HeLa-Cx43^GFP^/ACE2^mCherry^ under control conditions (top pannel) or after treatment with 50 pM spike S1 for 24 h (bottom pannel). (B) Averaged data normalized to control (dashed line) of SL/DT with DAPI by HeLa-Cx43^GFP^/ACE2^mCherry^ treated with 50 pM spike S1 for 24 h. *p < 0.05, the effect of spike S1 compared to control (two-tailed Student’s unpaired t test). Averaged data were obtained from three independent experiments with four repeats each. Scale bar = 100 μm

Cell-to-cell communication mediated by gap junctions is crucial for propagating intercellular Ca^2+^ waves to ensure proper cellular function and coordination [70]. Given that increased hemichannel opening induced by inflammatory conditions occurs along with the decrease in dye coupling [60], we investigated whether the functional state of gap junctions was affected by spike S1 in our setup. To examine gap junctional communication the scrape loading/dye transfer (SL/DT) technique was employed using DAPI. The treatment with 50 pM spike S1 for 24 h decreased in ~ 55% DAPI diffusion in Hela-Cx43^GFP^/ACE2^mCherry^ cells compared to control conditions (Fig. 4A and **B**). These results suggest that spike S1 oppositely regulates the activity of Cx43 hemichannels and gap junction channels.

### Spike S1 elevates the release of ATP and ATP-dependent Ca^2+^ dynamics via Cx43 hemichannels: potentiation by ACE2

**Fig. 5:**
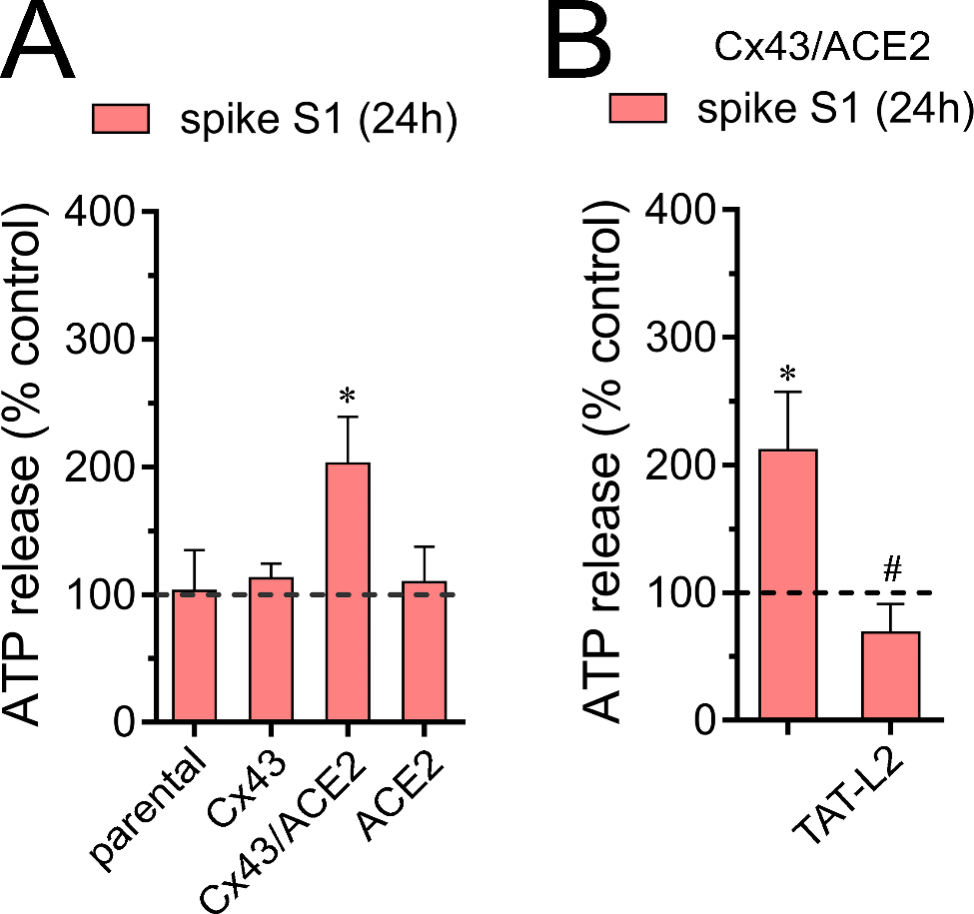
Spike S1 elevates the release of ATP via Cx43 hemichannels. (**A**) Averaged data of ATP release normalized with the control condition (dashed line) by HeLa-parental, HeLa-Cx43^GFP^, HeLa-Cx43^GFP^/ACE2^mCherry^ , or HeLa-ACE2^mCherry^ cells, treated for 24 h with 50 pM spike S1. *p < 0.05, the effect of spike S1 compared to control (one-way ANOVA followed by Tukey’s post-hoc test). (**B**) Averaged data of ATP release normalized with the control condition (dashed line) by HeLa-Cx43^GFP^/ACE2^mCherry^ cells stimulated with 50 pM spike S1 for 24 h alone or plus 200 µM TAT-L2. *p < 0.05, the effect of spike S1 compared to control; ^#^p < 0.05; effect of spike S1 vs. blockers 1 (one-way ANOVA followed by Tukey’s post-hoc test). Data were obtained from at least three independent experiments with four repeats each

Extracellular ATP plays a crucial role as a signaling molecule during various inflammatory conditions, attracting both innate and acquired immune systems to the site of injury or infection [71]. Cx43 hemichannels are permeable to ATP, as demonstrated by its bioluminescence imaging combined with single-channel recordings [68]. Relevantly, the release of ATP through Cx43 hemichannels has been shown to trigger immune cell activation and inflammation, contributing to the inflammatory response [72–74]. In this context and given that SARS-CoV-2 induces the release of ATP [65, 75], we examined whether spike S1 could affect the release of this messenger in our experimental setup. Treatment with 50 pM spike S1for 24 h did not modify the release of ATP in HeLa-Cx43^GFP^ cells (Fig. 5A). Similar findings were observed in HeLa-parental or HeLa-ACE2^mCherry^ cells stimulated with spike S1 (Fig. 5A). Interestingly, HeLa-Cx43^GFP^/ACE2^mCherry^ cells treated with 50 pM spike S1 for 24 h showed a significant 2-fold increase in the release of ATP compared to control conditions (Fig. 5A). This response was inhibited entirely to control values with the specific Cx43 hemichannel blocker TAT-L2 (Fig. 5B). These results suggest that spike S1 induces the release of ATP by a mechanism implicating the activation of Cx43 hemichannels and ACE2.

**Fig. 6:**
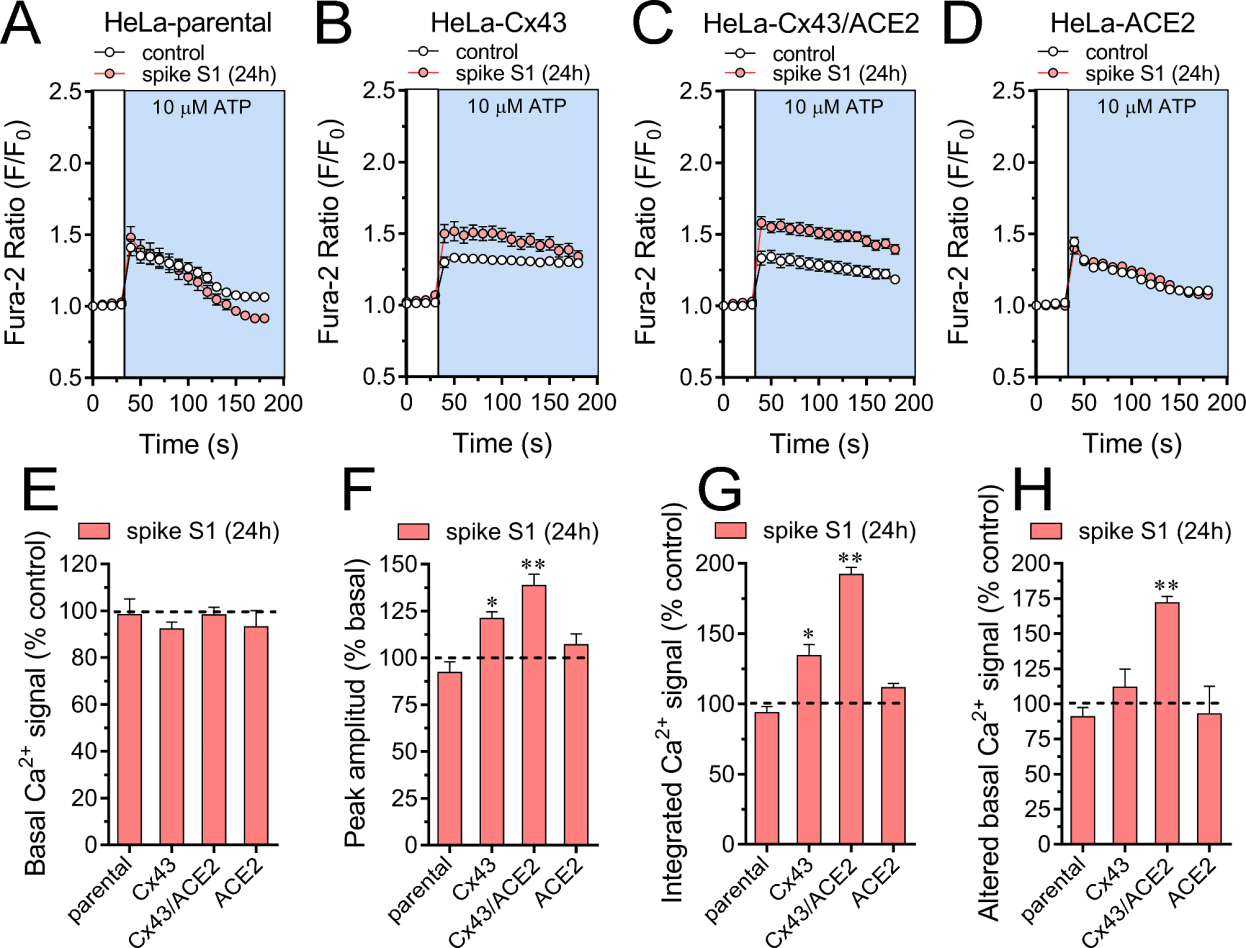
Spike S1 augments ATP-dependent Ca^2+^ dynamics via Cx43 hemichannels. (**A-D**) Representative plots of relative changes in Ca^2+^ signal over time induced by 100 µM ATP (light blue background) in HeLa-parental (**A**), HeLa-Cx43^GFP^ (**B**), HeLa-Cx43^GFP^/ACE2^mCherry^ (**C**) or HeLa-ACE2^mCherry^ (**D**) cells under control conditions (white circles) or treated with 50 pM spike S1 for 24 h (red circles). (**E-H**) Averaged data normalized to the control conditions (dashed line) of basal Ca^2+^ signal (**E**), ATP-induced peak amplitude normalized to basal Fura-2 ratio (**F**), integrated ATP-induced Fura-2 ratio response (**G**) and altered basal Fura-2 ratio (**H**) of HeLa-parental, HeLa-Cx43^GFP^, HeLa-Cx43^GFP^/ACE2^mCherry^ or HeLa-ACE2^mCherry^ treated with 50 pM spike S1 for 24 h. *p < 0.05, **p < 0.01; effect of spike S1 compared to control (one-way ANOVA followed by Tukey’s post-hoc test). Data were obtained from at least three independent experiments with two repeats each (≥ 12 cells analyzed for each repeat)

The activity of Cx43 hemichannels increases in response to moderate increases (> 500 nM) in [Ca^2+^]_i_ [76]. More relevantly, these channels can potentially allow extracellular Ca^2+^ to flow into cells, thereby playing a crucial role in maintaining [Ca^2+^]_i_ homeostasis [77, 78]. A recent report indicated that spike S1 elicits Ca^2+^ influx in pancreatic stellate cells and macrophages [79]. In this scenario, we tested whether spike S1 could alter [Ca^2+^]_i_ levels in our system (Fig. 6A-D). Fura-2 ratio (340/380) time-lapse recordings showed that basal [Ca^2+^]_i_ in all HeLa transfectants remained unchanged upon treatment with 50 pM spike S1 for 24 h (Fig. 6E). Although spike S1 did not affect the basal [Ca^2+^]_i_, it may still impact [Ca^2+^]_i_ dynamics triggered by paracrine molecules. Because we found that spike S1 triggers the release of ATP via Cx43 hemichannels, we investigated the effect of spike S1 on [Ca^2+^]_i_ responses elicited by this paracrine messenger. When HeLa parental or HeLa-ACE2^mCherry^ cells were acutely stimulated with 10 µM ATP, a rapid [Ca^2+^]_i_ peak was observed, followed by a quick return to basal levels (Fig. 6A and **D**). Spike S1 did not modify the ATP-mediated [Ca^2+^]_i_ responses in these cells (Fig. 6A and **D-H**). In contrast, Hela-Cx43^GFP^ or HeLa-Cx43^GFP^/ACE2^mCherry^ cells showed a rapid [Ca^2+^]_i_ peak upon ATP stimulation with slow returning kinetics to basal values (Fig. 6B and **C**). Notably, spike S1 increased the peak amplitude and integrated [Ca^2+^]_i_ signal triggered by ATP in Hela-Cx43^GFP^ cells (Fig. 6B and **F-H**). These ATP-mediated [Ca^2+^]_i_ responses were potentiated in HeLa-Cx43^GFP^/ACE2^mCherry^ cells treated with spike S1 (Fig. 6C and **F-H**). Altogether these findings suggest that the opening of Cx43 hemichannels contributes to the spike S1-induced augment in ATP-mediated [Ca^2+^]_i_ dynamics, this response being potentiated with the co-presence of ACE2.

**Fig. 7:**
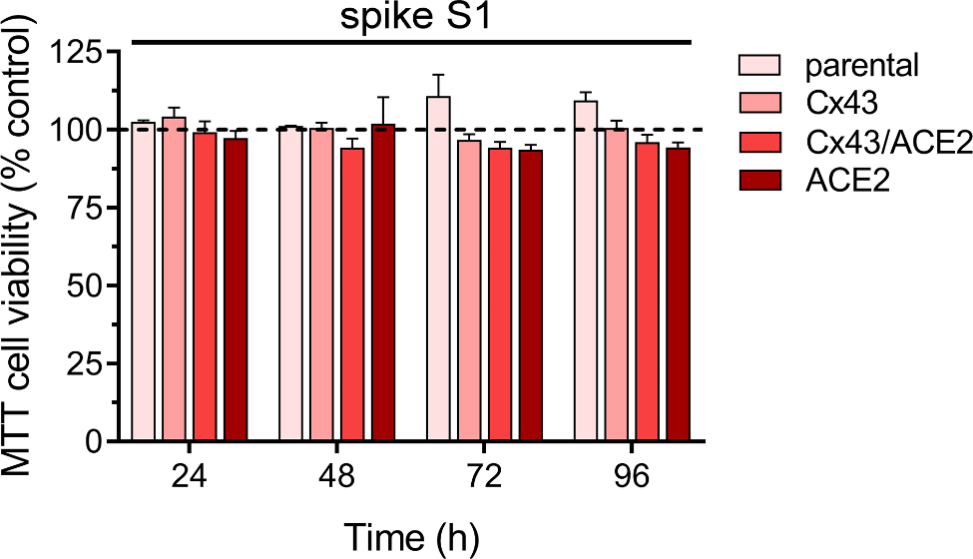
Spike S1 has no effect on cell viability. Averaged data normalized to the control conditions (dashed line) of MTT cell viability by HeLa-parental, HeLa-Cx43^GFP^, HeLa-Cx43^GFP^/ACE2^mCherry^ or HeLa-ACE2^mCherry^ treated with 50 pM spike S1 for 24, 48, 72 or 96 h. Data were obtained from at least three independent experiments with two repeats each

Recent studies have reported that spike S1 induces apoptosis in various cell types [80–82], while prolonged activation of Cx43 hemichannels has been shown to reduce cell viability under pathological conditions [53, 83, 84]. Given this context, we investigated whether spike S1 could impact cell viability in our experimental setup. To assess this, we measured cell viability by quantifying the reduction of MTT to formazan, a process directly correlated with the number of metabolically active cells in culture. Interestingly, treatment with spike S1 for varying periods of stimulation did not result in any changes in cell viability across all HeLa cell transfectants, parental, Cx43^GFP^, HeLa-Cx43^GFP^/ACE2^mCherry^ or ACE2^mCherry^ (Fig. 7). These data suggest that under these conditions, spike S1 has no significant impact on cell survival.

## Discussion

This study reveals for the first time that spike S1 enhances the activity of Cx43 hemichannels. The presence of the SARS-CoV-2 binding receptor ACE2 potentiated this effect. Interestingly, the spike S1-induced opening of Cx43 hemichannels leads to significant changes in [Ca^2+^]_i_ dynamics and ATP release. By performing time-lapse recordings, we observed that spike S1 time and dose-dependently increased the uptake of Etd in HeLa-Cx43^GFP^ but not HeLa-parental cells. To test whether ACE2 could contribute to the spike S1-induced activity of Cx43 hemichannels, we further performed time-lapse recordings of DAPI uptake in HeLa-Cx43^GFP^/ACE2^mCherry^ cells. The expression of ACE2^mCherry^ did not alter the functional opening of Cx43 hemichannels, as DCFS media triggered similar increments in DAPI uptake in HeLa-Cx43^GFP^ and HeLa-Cx43^GFP^/ACE2^mCherry^ cells. As expected, no changes in DAPI uptake were detected in HeLa-parental or HeLa-ACE2^mCherry^ cells. Remarkably, DAPI uptake evoked by spike S1 in HeLa-Cx43^GFP^ cells was potentiated when similar experiments were conducted on HeLa-Cx43^GFP^/ACE2^mCherry^ cells.

The involvement of Cx43 hemichannels in spike S1-induced DAPI uptake in HeLa-Cx43^GFP^/ACE2^mCherry^ cells was confirmed using selective mimetic peptides (gap19 and TAT-L2) to antagonize Cx43 hemichannels. The latter was consistent with the fact that blocking Panx1 hemichannels with ^10^panx1 did not elicit any inhibitory effect. Electrophysiological experiments in whole-cell configuration further supported these findings, as both La^3+^ and high extracellular Ca^2+^ completely suppressed the Cx43 hemichannel currents produced by spike S1. These results align with previous studies demonstrating that viruses or their viral proteins trigger the activation of Cx43 hemichannels [49, 50]. Numerous lines of evidence indicate that during inflammatory scenarios, hemichannels and gap junction channels in exhibit contrasting activation patterns [60]. Supporting this inverse regulation, we observed that the opening of Cx43 hemichannels induced by spike S1 coincided with a reduction in cell-cell coupling in HeLa Cx43^GFP^/ACE2^mCherry^ cells, as evidenced by the intercellular diffusion of DAPI.

Previous research has demonstrated that SARS-CoV-2 induces the release of various “danger” signals, including ATP [65, 75]. The release of ATP occurs through Cx43 hemichannels [68], which could amplify cell damage by impairing [Ca^2+^]_i_ dynamics and [Ca^2+^]_i_ homeostasis [42, 85–87]. Here, we found that activation of Cx43 hemichannels and ACE2 expression were crucial for the spike S1-induced release of ATP. These findings are consistent with previous studies highlighting the release of ATP via Cx43 hemichannels in cells treated with viruses or their viral proteins such as HIV and gp120 [49, 50]. P2Y receptor-dependent release of Ca^2+^ from internal stores and extracellular Ca^2+^ influx via P2X receptors are characteristic cellular responses to ATP [88]. We found that while spike S1 did not affect basal levels of [Ca^2+^]_i_, it significantly increased ATP-induced [Ca^2+^]_i_ responses, including signal amplitude, the integrated area under the curve, and sustained signal. Cx43 hemichannels were crucial for these responses as they only occurred on Cx43 HeLa transfectants but not parental HeLa cells. More relevantly, the expression of both Cx43 and ACE2 in HeLa cells largely potentiated this phenomenon. Furthermore, our findings indicate that spike S1 does not have an impact on cell viability. This suggests that spike S1-mediated opening of Cx43 hemichannels and further release of ATP primarily impair cell function rather than directly causing cell death.

What are the mechanisms behind the activation of Cx43 hemichannels evoked by spike S1? They could be multiple. For example, acute stimulation with spike S1 leads to an ACE2-dependent persistent increase in [Ca^2+^]_i_ baseline in pulmonary endothelial cells [89] and spontaneous [Ca^2+^]_i_ transients in pancreatic cells [79]. Since a moderate rise in [Ca^2+^]_i_ (< 500 nM) significantly enhances Cx43 hemichannel activity [76], the spike S1-mediated augment of Cx43 hemichannel activity could rely on [Ca^2+^]_i_. The S-nitrosylation of Cx43 hemichannels by the action of nitric oxide (NO) could be another possibility to explain the opening of these channels. Indeed, the spike protein S1 has been shown to enhance the release of NO in microglia [90]. Additionally, it triggers the production of IL-1β and TNF, along with the activation of p38 MAP kinase via Toll-like receptor 4 [90]. Importantly, these factors are well-documented for their role in promoting the opening of Cx43 hemichannels during pro-inflammatory conditions [91, 92]. In this context, we speculate that the Cx43 hemichannel-mediated release of ATP and/or its derivatives (e.g., ADP) may propagate the signaling effects of spike S1 to neighboring cells, leading to [Ca^2+^]_i_ responses that can impair cell function. More relevantly, given that Cx43 hemichannels are permeable to Ca^2+^ [78] they could contribute to perpetuating the propagation of ATP-mediated signaling.

Could the spike S1-induced activation of Cx43 hemichannels potentially account for the side effects on SARS-CoV-2 vaccines? The S protein-encoding mRNA vaccines targeting SARS-CoV-2 trigger enduring and robust systemic immunity, and thus, their contribution to stemming the COVID-19 pandemic and safeguarding countless lives is indisputable. While uncommon, adverse effects of mRNA vaccines encompass acute myocardial infarction, Bell’s palsy, cerebral venous sinus thrombosis, Guillain–Barré syndrome, myocarditis/pericarditis, and severe clinical conditions [93, 94]. In addition to the potential allergic reactions of lipid nanoparticles [95] and packaged mRNA [96], adverse effects resulting from vaccination may be linked to distinctive properties of the S protein itself, either due to molecular mimicry with human proteins or its role as an ACE2 ligand. With this in mind, it is possible to speculate that the opening of Cx43 hemichannels and their downstream signaling could account for the adverse effect in cellular function evoked by spike S1 acting on ACE2 receptors. Further research is required to clarify the potential involvement of Cx43 hemichannels in this context.

## Conclusions

Our findings indicate that upon SARS-CoV-2 infection, activation of Cx43 hemichannels by spike S1 occurs rapidly. This activation leads to an increase in the release of ATP and enhances ATP-mediated [Ca^2+^]_i_ dynamics. We speculate that this response may be amplified in cells expressing high levels of ACE2, particularly those found in the cardiorespiratory system [97]. Our study suggests a novel mechanism through which SARS-CoV-2 disrupts cell function. This mechanism could involve the successive release of ATP and Cx43 hemichannel-mediated [Ca^2+^]_i_ signaling via spike protein S1. Understanding the molecular mechanisms underlying these effects could unveil potential pharmacological targets for developing therapies to counteract SARS-CoV-2 infection and related diseases, including Long-COVID.

## Methods

### Reagents and antibodies

HEPES, ATP, bzATP, NaCl, KCl, CaCl_2_, MgCl_2_ and glucose were obtained from MERCK (Darmstadt, Germany). Penicillin (10000U) and streptomycin (10 mg/mL) were purchased from Sigma-Aldrich (St. Louis, MO, USA). Fetal bovine serum (FBS) was purchased from Hyclone (Logan, UT, USA), whereas Trypsin 10X, Hank’s solution, ATP determination kit, Fura-2AM, Dulbecco’s Modified Eagle Medium (DMEM), Phosphate-Buffered Saline (PBS) and Etd bromide (10 mg/mL) were purchased from Thermo Fisher Scientific (Waltham, MA, USA). The Recombinant human coronavirus SARS-CoV-2 Spike Glycoprotein S1 (ab273068, Wuhan-Hu-1 variant: MN908947) and 4′,6-diamidino-2-phenylindole (DAPI) were obtained from Abcam (Cambridge, UK). The mimetic peptides gap19 (KQIEIKKFK, intracellular loop domain of Cx43), TAT-L2 (YGRKKRRQRRR-DGANVDMHLKQIEIKKFKYGIEEHGK, intracellular loop domain of Cx43) and ^10^panx1 (WRQAAFVDSY, first extracellular loop domain of Panx1) were obtained from Genscript (New Jersey, USA).

### Cell cultures

HeLa cells (ATCC, Rockville, MD, USA) were cultured in DMEM with 10% FBS, 100 U/ml penicillin and 100 µg/ml streptomycin sulfate (Nunc, Roskilde, Denmark). Attached cells were dissociated for sub-culturing with 0.05% trypsin-EDTA (ThermoFisher Scientific, Massachusetts, USA). For single transfection, HeLa cells were seeded in 35 mm plates and grown at ~ 60% confluence. Then, they were transfected with 1 µg of pRP (Exp-mCherry/Bsd-CMV/hACE2 vector (NM-021804.3, VectorBuilder Inc, Chicago, IL, USA), or pCMV6-AC-Cx43^GFP^ (Origene, Origene, Rockville, MD, USA). Optimem medium (ThermoFisher Scientific, Waltham, MA, USA), plus Lipofectamine 2000 and 1 µg of the plasmids were mixed. Then, the culture medium was replaced with Optimem, and the transfection mixture was added. After 4 h, the cells were incubated in DMEM with 10% FBS at 37 °C, in 5% CO_2_, and 48 h later, they were evaluated for expression in an epifluorescence microscope. Selection started by adding the antibiotic G418 in the case of HeLa expressing Cx43^GFP^ or blasticidin in the case of HeLa cells expressing ACE2 to the culture media three times per week for two weeks. Parental or ACE2 HeLa cells do not show levels of Cx43 (**Supplementary Fig. 1**). In the case of the double transfected cells, selected HeLa Cx43^GFP^ cells were transfected with ACE2 following the same protocol described above, but in this case, cells were maintained with G418 and Bsd for two weeks or until a stable clone expressing GFP and mCherry were observed under the microscope. In our hands, after selection, ~ 70% of the cells were expressing both proteins.

### Cell treatments

Depending on the experiment and cell line type, cells were treated with 10, 50, 100 or 1000 pM Spike S1 at different periods of exposure: acutely or 1, 24, 48, or 72 h. To obtain conditioned media from cells, they were seeded (2 × 10^6^ cells in 35 mm dishes) in DMEM containing 10% FBS and treated with 50 pM spike S1 for 24 h. Supernatants were collected, filtered (0.22 μm), and stored at -20 °C before being used for experiments. Mimetic peptides against Cx43 (100 µM gap19 or TAT-L2) or Panx1 hemichannels (100 µM ^10^panx1) were used in cell cultures 15 min before and co-applied during dye uptake and time-lapse recordings (see below). The effectiveness of ^10^panx1 mimetic peptide was confirmed by its inhibitory effect on BzATP-induced Etd uptake in cultured astrocytes (**Supplementary Fig. 2**), a well-known stimulus that activates Panx1 hemichannels in these cells.

### Dye uptake and time-lapse fluorescence imaging

To characterize the functional state of hemichannels, dye uptake experiments using Etd and DAPI were performed. In the case of Etd uptake, cells were bathed with recording solution (in mM): 148 NaCl, 5 KCl, 1.8 CaCl_2_, 1 MgCl_2_, 5 glucose, and 5 HEPES, pH 7.4, containing 5 µM Etd and imaged on an Olympus BX 51W1I upright microscope. Etd fluorescence was captured every 30 s for 8 min by a Retiga 1300I fast-cooling monochrome digital monochrome camera (12-bit; Q Imaging, Burnaby, BC, Canada) controlled by Metafluor imaging software (Universal Imaging, Downingtown, PA, USA). In the case of DAPI uptake, cells were bathed with recording solution (in mM): 140 NaCl, 4 KCl, 2 CaCl_2_, 1 MgCl_2_, 5 glucose, and 10 HEPES, pH = 7.4 containing 10 µM DAPI and imaged on an inverted microscope (Eclipse Ti-U, Nikon). In some experiments, cells were exposed to a divalent cation-free solution (DCFS), which was comprised of (in mM): 140 NaCl, 4 KCl, 5 mM EGTA (an extracellular Ca^2+^ chelator), 5 glucose, and 10 HEPES, pH = 7.4. DAPI fluorescence intensity was captured every 20 s during a 20 min period at room temperature, using a light LED-based source and adequate filters (Ex/Em) for GFP, mCherry, and DAPI. NIS element advanced research software (version 4.0, Nikon) was used for data acquisition and image analysis. Fluorescence intensity was recorded in 15–20 cells with ImageJ software and calculated with the following formula: corrected total cell fluorescence = integrated density - ([Selected cell area] × [Mean fluorescence of background readings]). The average slope of the fluorescence ratio during a given time interval (ΔF/ΔT) was calculated using Excel and GraphPad Prism software, where it will be expressed as the Etd uptake rate (AU/min).

### Electrophysiology

HeLa cells were grown in a 60 mm plastic dish until 60–70% confluence. The day of experimentation, cells were washed with recording solution twice. Then, 1 ml of 0.05% trypsin-EDTA (ThermoFisher Scientific, Massachusetts, USA) was added, and cells were placed at 37 °C for approximately 1 min or until cells looked partially detached. At this point, cells were gently detached using a 1 ml micropipette. Cells were placed in a 15 plastic tube containing 5 ml DMEM plus 10% FBS and centrifuged for 3 min at 1300 rpm. Then, the supernatant was discharged, and cells were resuspended in 6 ml of recording media and centrifuged at 1300 rpm for 3 min. This step was repeated twice. Finally, cells were resuspended in 200 ml recording solution and placed in a 1.5 ml Eppendorf tube. Following a recovery period (30 min at room temperature), cells were gently resuspended and 8 µl of cell suspension was placed in the Patchliner (NPC-16 Patchliner system, Nanion Technologies GmbH, Germany). Patch-control HT software (HEKA Elektronik, Germany) was used to control the pressure necessary to establish the whole-cell configuration. Hemichannel currents were recorded at room temperature (22–23 °C), using an internal solution (in mM): 10 NaF, 110 CsF, 20 CsCl, 2 EGTA, and 10 HEPES, pH 7.4 (adjusted with CsOH) and external solution equal to recording solution described in the previous section. Following cell contact with the 3–5 MΩ planar electrode, 30 µl of seal enhancer solution (in mM: 80 NaCl, 3 KCl, 35 CaCl_2_, 10 HEPES/NaOH pH 7.4) were added to the external solution to promote giga-ohm seal formation. After establishing the whole-cell configuration, the seal enhancer solution was replaced with two washes with a recording solution. Cells were allowed to stabilize for 2 min after starting whole-cell recordings. Patchmaster software (HEKA) was used to automatically compensate for whole-cell capacitance and series resistance, and perform voltage-clamp protocols. Igor Pro 9 was used to analyze the data and create the figures.

### Scrape loading/dye transfer assay

Gap junction permeability was evaluated at room temperature using the scrape-loading/dye transfer (SL/DT) technique. Briefly, cells cultures were washed for 10 min in HEPES-buffered salt solution containing the following (in mM): 140 NaCl, 5.5 KCl, 1.8 CaCl_2_, 1 MgCl_2_, 5 glucose, 10 HEPES, pH 7.4 followed by washing in a Ca^2+^-free HEPES solution for 1 min. Then, a razor blade cut was made in the monolayer in a HEPES-buffered salt solution with normal Ca^2+^ concentration containing 10 µM DAPI. After 3 min, DAPI was washed out several times with HEPES-buffered salt solution. At 15 min after scraping, fluorescent images were captured using a a Zeiss Axio Observer D.1 Inverted Microscope with a Solid-State Colibri 7 LED illuminator and with a 20x objective. Changes were monitored using an AxioCam MRm monochrome digital camera R3.0 (Carl Zeiss AG, Zeiss, Oberkochen, Germany), and Software ZEN Pro (Zen 2.3 [blue edition], Carl Zeiss AG, Oberkochen, Germany) for image acquisition and analysis. For each trial, data were quantified by measuring fluorescence areas in three representative fields. Quantification of changes in gap junctional communication induced by different treatments was performed by measuring the fluorescence area, expressed as arbitrary units (AU).

### [Ca^2+^]_i_ cell imaging

Cells plated on glass coverslips were loaded with 5 µM Fura-2-AM in DMEM without serum at 37 °C for 45 min and then washed three times in recording solution (in mM): 140 NaCl, 4 KCl, 2 CaCl_2_, 1 MgCl_2_, 5 glucose, and 10 HEPES, pH = 7.4, followed by de-esterification at 37 °C for 15 min. The experimental protocol for Ca^2+^ signal imaging involved data acquisition every 10 s for 5 min (emission at 510 and 515 nm, respectively) at 340/380-nm excitation wavelengths (Xenon lamp) using an inverted microscope (Eclipse Ti-U, Nikon). NIS element advanced research software (version 4.0, Nikon) was used for data acquisition and image analysis. Fluorescence intensity recorded in 15 cells involved the determination of pixels assigned to each cell. The average pixel value allocated to each cell was obtained with excitation at each wavelength and corrected for background. The Fura-2 ratio was obtained after dividing the 340-nm by the 380-nm fluorescence image on a pixel-by-pixel base (R = F_340 nm_/F_380 nm_).

### Measurement of ATP

The extracellular amount of ATP was measured using the ATP determination kit according to the protocol provided by its supplier Invitrogen (ATP Determination Kit, A22066). Using the conditioned medium of the different treated cell lines and applying the kit that includes D-Luciferin and recombinant firefly luciferase, the luminescence produced by luciferin upon binding with ATP present in the conditioned medium was measured using a Tecan Infinite® M200 PRO plate reader spectrometer (Männedorf, Switzerland) and the i-control™ software.

### Assessment of cell viability

Cells seeded at 5 × 10^4^ cells/well in 96-well plate were treated or not (control) with spike S1 for 24, 48, 72 or 96 h. 10 µl of aqueous MTT solution (4 mg/ml) was then added to each well (100 µl), and the mixture was incubated at 37 °C for 3 h. The MTT solution was carefully decanted off, and formazan was extracted from the cells with 100 µl of a 4:1 DMSO–EtOH mixture in each well. Color was measured with a 96-well ELISA plate reader at 550 nm, with the reference filter set to 620 nm. All MTT assays were repeated three times.

### Western blot

Hela cells were lysed in 200 µl of RIPA lysis buffer supplemented with complete protease inhibitors (Roche) and sonicated on ice. Protein concentration was quantified using the Qubit Protein assay kit (Life Technologies) and the Qubit 3.0 Fluorometer (ThermoFisher Scientific). A total of 50 µg of denatured proteins in loading buffer were prepared by heating at 95 °C for 3 min. At this point, proteins were separated by 10% SDS-PAGE gel electrophoresis or stored at -80 °C. After the electrophoresis, proteins were transferred to 0.45 nm nitrocellulose membranes (Bio-Rad) using the Mini-PROTEAN Tetra System (Bio-Rad). Then, membranes were blocked with 5% milk in 0.05% TBST for at least 30 min at room temperature. After that, membranes were incubated with primary antibodies diluted in a blocking solution overnight. In the next day, membranes were washed three times with 0.05% TBST, following the incubation with secondary antibodies in a blocking solution for 2 h at room temperature. To finish, membranes were washed three times with 0.05% TBST and exposed to Luminata Forte HRP substrate (Millipore) and visualized with the C-Digit Chemiluminescence Western Blot Scanner system ECL (LI-COR, Inc, Lincoln, USA).

### Data analysis and statistics

Results were expressed as mean ± standard error of the mean (SEM); *n* refers to the number of independent experiments performed. Statistical analysis was performed using GraphPad Prism (version 9, GraphPad Software, La Jolla, CA, USA). Normality and equal variances were assessed by the Shapiro-Wilk normality test and Brown-Forsythe test, respectively. Depending on the nature of the data, they were analyzed with parametric or nonparametric tests. When the data were normal, without unequal variance and non-heteroscedastic, a t-test was used to compare two groups and in case of multiple comparisons, one or two-way analysis of variance (ANOVA) was used followed, in the case of significance, of a Tukey’s post-hoc test. When the data were heterocedastic as well as non-normal and with unequal variation, nonparametric tests were used, such as Kruskal-Wallis test followed, in the case of the significance, by a Dunn’s post-hoc test. Details of the statistical results, together with the n and number of replicates, are included in figure legends. A probability of p < 0.05 was considered statistically significant.

### Electronic supplementary material

Below is the link to the electronic supplementary material.

Supplementary Material 1

Supplementary Material 2

## Data Availability

The datasets used and/or analyzed during the current study are available from the corresponding author upon reasonable request.

## Abbreviations

ACE2: Angiotensin-converting enzyme 2
COVID-19: Coronavirus disease 2019
Cx43: Connexin 43
DCFS: Divalent cation-free solution
DMEM: Dulbecco’s Modified Eagle Medium
Etd: Ethidium
FBS: Fetal bovine serum
HIV: Human immunodeficiency virus
Panx1: Pannexin-1
PBS: Phosphate-Buffered Saline
SARS-CoV-2: Severe acute respiratory syndrome coronavirus 2
Spike S1: Spike protein S1
