# Supplementary figures and images for: SARS-CoV-2 spike protein S1 activates Cx43 hemichannels and disturbs intracellular Ca2+ dynamics
Source: Biol Res. 2023 Oct 25;56:56. doi: 10.1186/s40659-023-00468-9 (PMC10598968; doi:10.1186/s40659-023-00468-9)

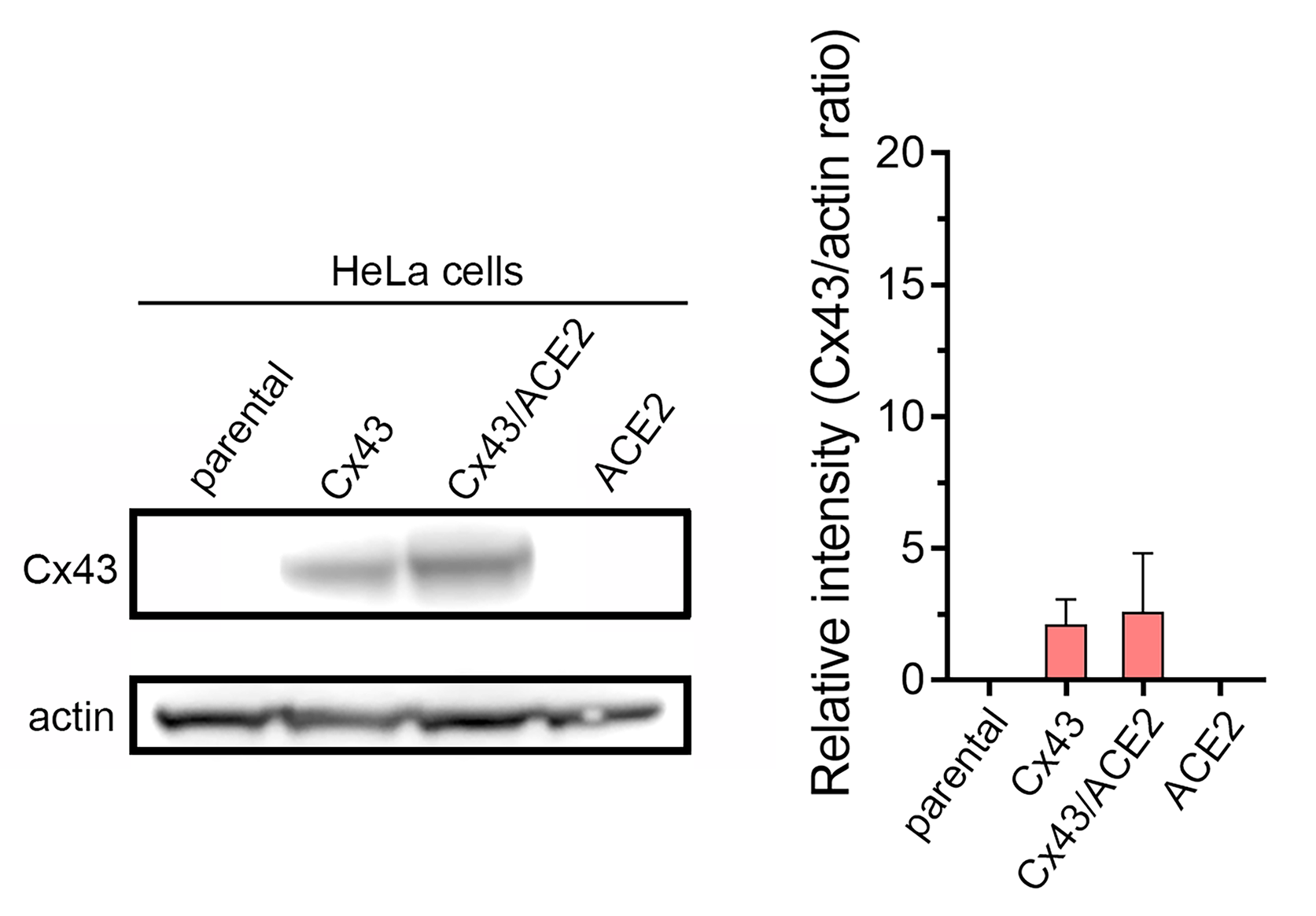

Supplement: Supplementary file 1 — Supplementary Material 1 [file 40659_2023_468_MOESM1_ESM.tif]

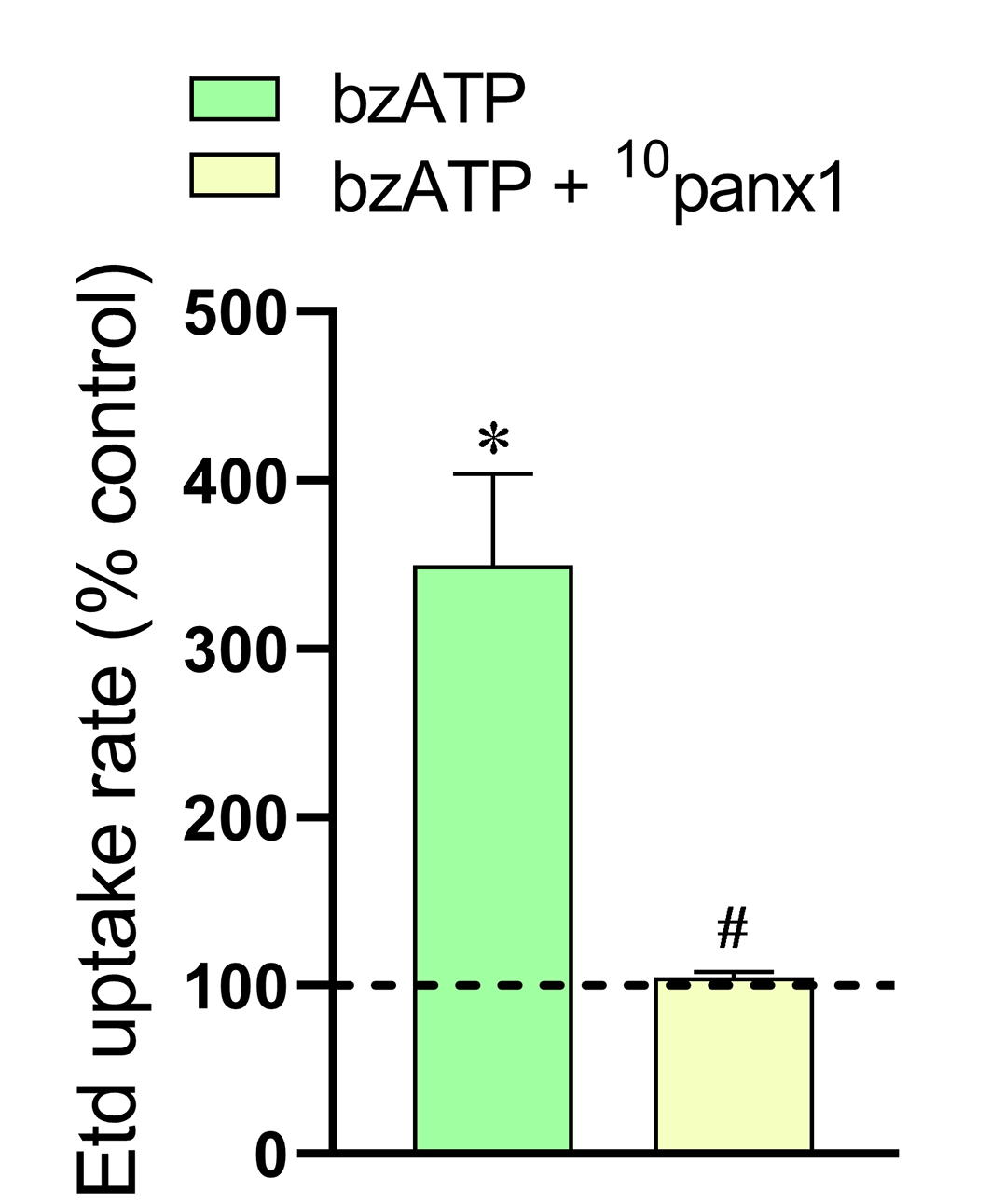

Supplement: Supplementary file 2 — Supplementary Material 2 [file 40659_2023_468_MOESM2_ESM.tif]
